# Fetal bladder rupture after high-dose maternal opioid treatment: a case report

**DOI:** 10.1515/crpm-2023-0034

**Published:** 2024-04-29

**Authors:** Julia Herken, Vincent Uerlings, Sabine Zundel, Jonathan Aichner, Markus Hodel

**Affiliations:** Department of Obstetrics and Gynecology, Lucerne Cantonal Hospital, Luzern, Switzerland; Department of Pediatric Surgery, Children’s Hospital Lucerne, Luzern, Switzerland

**Keywords:** fetal bladder rupture, fetal urinary ascites, opioids in pregnancy

## Abstract

**Objectives:**

Fetal bladder rupture is rare and mainly caused by lower urinary tract obstruction (LUTO). Our case report describes a rupture at a gestational age of 31 weeks following high-dose maternal opioid exposure during intensive care treatment. Opioids perturb the interplay of afferent and efferent signals between the bladder, urethra, and the central nervous system (CNS) which is crucial in contributing to urinary retention. They rapidly cross the human placenta, affecting also the fetus. To date, there is no clear proof of the connection between maternal opioid treatment and fetal bladder rupture, but the association seems to strengthen.

**Case presentation:**

A 18-year old first Gravida at 31 weeks of gestation developed a severe sepsis with progressive hypoxic lung failure and need for intubation. During the ICU-treatment, several opioids were administered for sedation and pain relief. Four days after induction of opioid treatment the ultrasound revealed a decompressed fetal bladder, hematoma and significant ascites. Fetal bladder rupture with urinary ascites was suspected. A caesarean section was performed at 33 weeks of gestation due to massive fetal urinary ascites, fetal deterioration and imminent abdominal compartment syndrome. Adequate ventilation and circulation could only be established after percutaneous drainage of 350 mL of abdominal fluid, that was confirmed to be urine. A defect of the bladder was confirmed by ultrasound. On the fifth day of life, the bladder was closed surgically by pediatric surgery.

**Conclusions:**

Growing awareness of the possible connection between maternal opioid therapy and fetal bladder rupture is necessary to plan follow-up ultrasound examinations to assess the fetal situation.

## Introduction

Fetal bladder rupture is a very rare condition usually caused by congenital lower urinary tract obstruction (LUTO) [1]. However, this complication can exceptionally occur in the absence of any urogenital malformation after maternal high-dose opioid therapy [2], [3], [4], [5].

We report a case of opioid treatment related spontaneous fetal bladder at 31 weeks of gestation, which eventually led to premature delivery because of a deteriorating fetal condition.

## Case presentation

**Figure 1:**
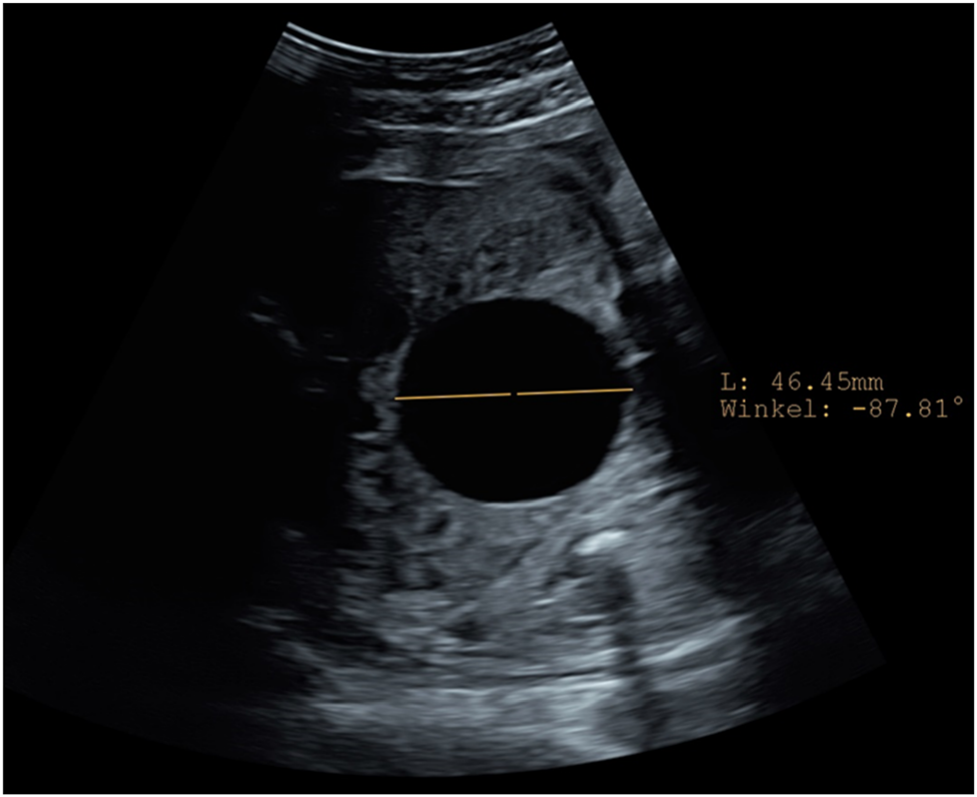
Two days after introduction of opioid treatment ultrasound showing an isolated enlarged fetal bladder (diameter of 47 × 40 mm) with thin walls.

**Figure 2:**
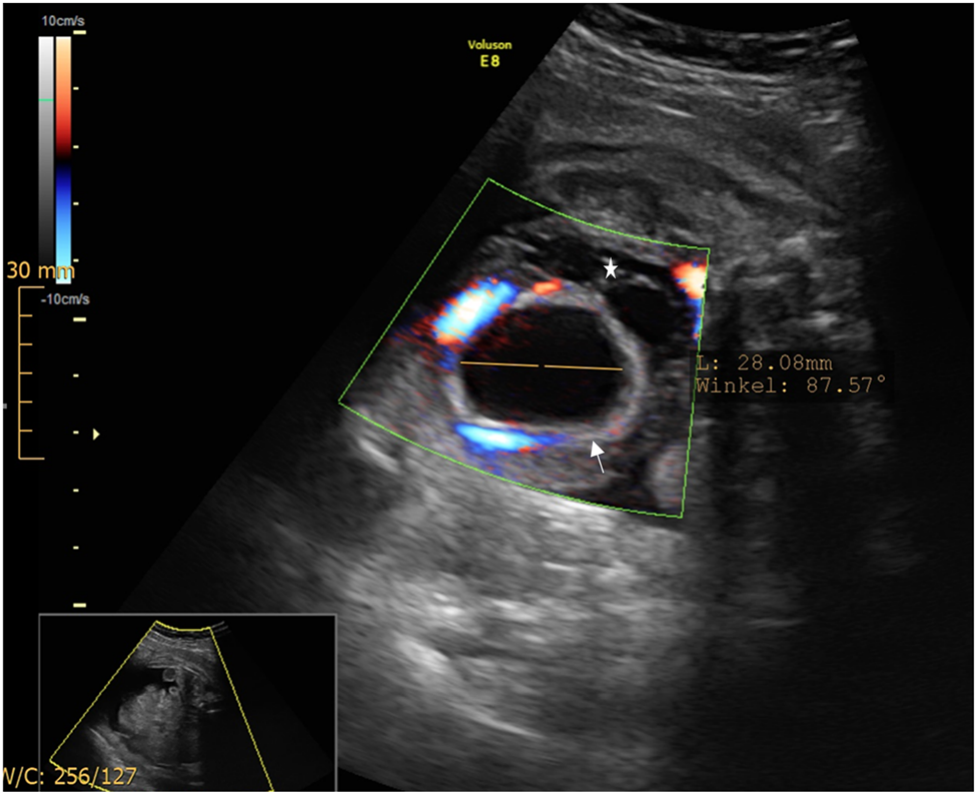
Ultrasound showing a decompressed fetal bladder (28 mm) with thickened walls (↑) and prevesical hematoma (*).

**Figure 3:**
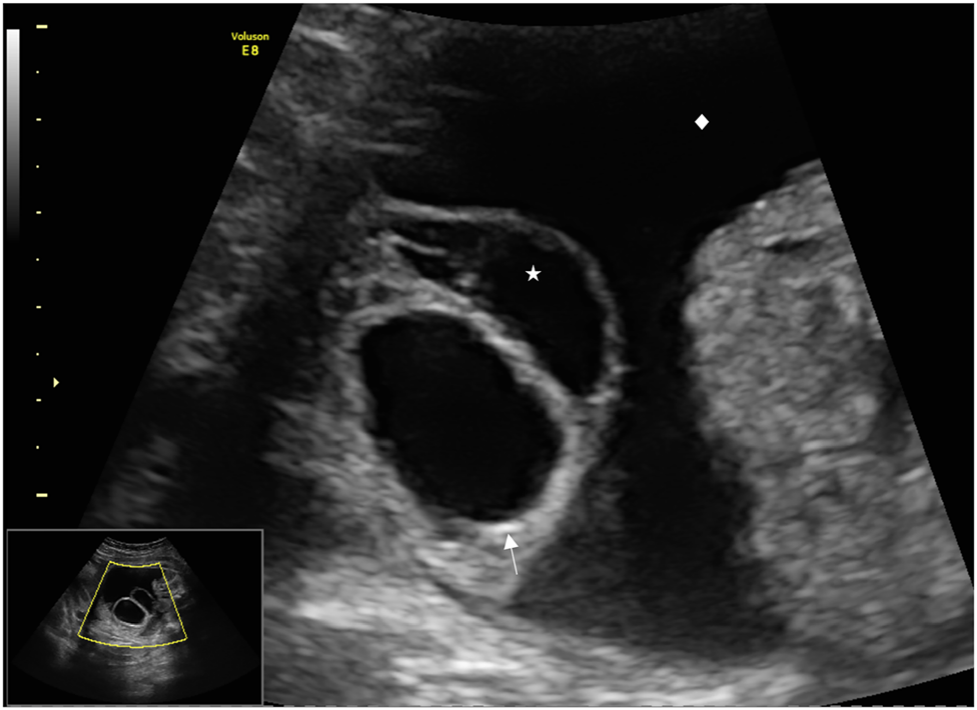
Fetal bladder (↑) rupture with suspected prevesical hematoma (*) and increasing urinary ascites (♦).

**Figure 4:**
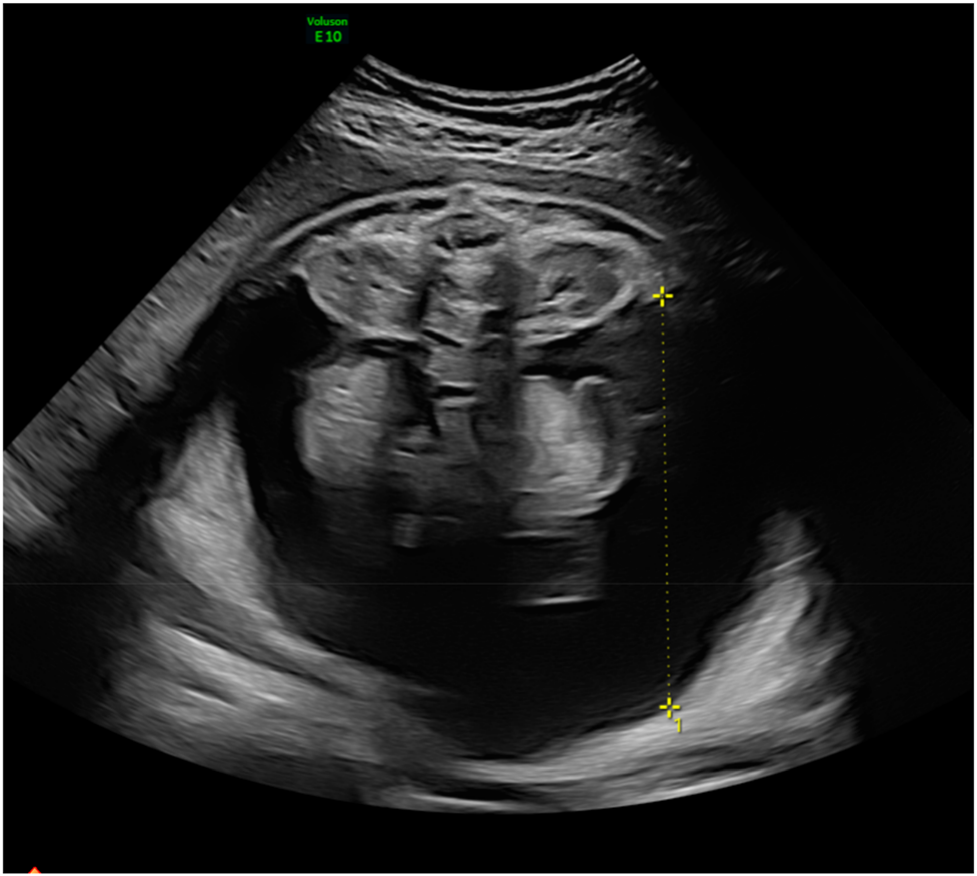
Massive urinary ascites with imminent compartment syndrome on day nine after suspected bladder rupture.

**Figure 5:**
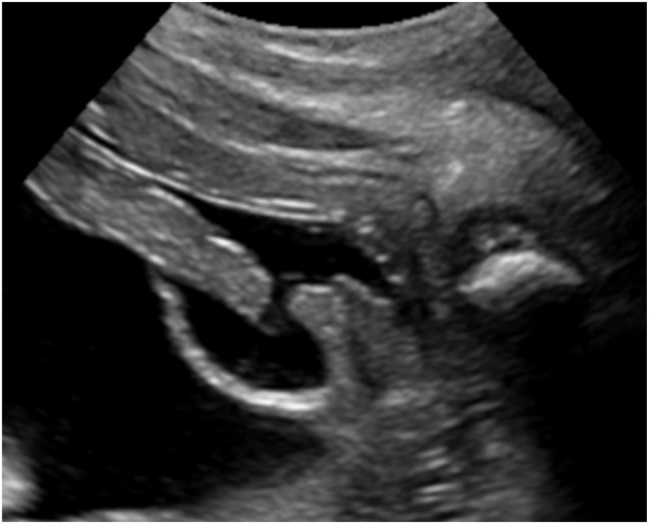
4th postpartal hour: ultrasound displaying a collapsed bladder with dorsal wall-defect, adherend web (peritoneum) and abdominal ascites.

A 18-year old first Gravida at 31 weeks of gestation developed a severe sepsis caused by an obstructive pyelonephritis. She was referred due to progressive hypoxic lung failure with need for intubation. The patient was transferred to an intensive care unit (ICU) and lung protective ventilation was carried out. Fetal monitoring was normal and showed a eutrophic, male, hemodynamically compensated fetus with a normal amount of amniotic fluid. No fetal morphological abnormalities were observed. Corticosteroid prophylaxis to prevent neonatal distress syndrome and magnesium sulfate for fetal neuroprotection in the event of a possible preterm delivery were applied. During the ICU-treatment, several opioids (remifentanil 10 ug/min for 48 h, fentanyl boluses and nalbuphine), propofol at a rate of 300 mg/h and the benzodiazepine midazolam as boluses of 2 mg were administered for sedation and pain relief over six days. Two days after introduction of opioid treatment, an isolated enlarged fetal bladder was observed (diameter of 47 × 40 mm) (Figure 1). Four days after induction of opioid treatment the ultrasound revealed a decompressed fetal bladder with a thickened wall, hematoma and significant ascites (Figures 2 and 3). The fetal kidneys were morphologically normal. Amniotic fluid and fetal monitoring were unremarkable. Because of the findings, a possible spontaneous bladder rupture with urinary ascites was suspected. After an interdisciplinary discussion, an expectant approach was decided on. The patient was closely monitored with daily ultrasound and cardiotocography. These revealed a constant increase of fetal ascites, while the amount of amniotic fluid decreased (Figure 4). Nine days after the suspected bladder rupture fetal doppler velocimetry showed progressive deterioration (cerebroplacental ratio (CPR) below 5th percentile, but no alterations in the ductus venosus flow). An anhydramnios was detected and fetal ascites reached such an extent that an abdominal compartment syndrome was imminent. After another interdisciplinary discussion it was decided that a preterm delivery was the safest option for the child. At 32 weeks and 2 days gestation caesarean delivery was performed. The initial aim was to provide care for the baby on a “Concord Birth Trolley” before cutting the umbilical cord, ensuring continued blood circulation through the mother, but the placenta detached immediately after the delivery. The child did not adapt (APGAR 1/5/7) and immediate mechanical resuscitation was necessary. Adequate ventilation and circulation could only be established after percutaneous drainage of 350 mL of abdominal fluid. Laboratory results confirmed the abdominal fluid to be urine. A dorsolateral defect of the bladder was confirmed by ultrasound (Figure 5). Non-operative management failed: the volume of the abdominal ascites increased constantly, and blood creatinine levels remained high (>45 ymol/L). On the fifth day of life, the bladder was closed surgically by pediatric surgery. The neonate recovered well. Cysto-urethrography showed a regular bladder, ruled out posterior urethral valves and regular voiding pattern has been observed ever since catheter removal.

## Discussion

Fetal bladder rupture causing urinary ascites is rare. A fetal megacystis might precede a rupture. In the first trimester, megacystis is defined as a longitudinal bladder diameter of ≥7 mm, and is present in 0.06 % of pregnancies [6]. The prevalence of megacystis beyond the first trimester remains unclear and there is no accepted definition [7]. The main cause of fetal megacystis, diagnosed in any trimester of pregnancy, is due to lower urinary tract obstruction (LUTO) [1]. In the male fetus, the most common diagnose are posterior urethral valves, female fetuses are hardly ever affected. The rare Megacystis-Microcolon-Intestinal Hypoperistalsis Syndrome (MMIHS) is also accompanied by a significantly enlarged urinary bladder. It predominantly affects female fetuses. A megacystis can also be present as a concomitant finding of genetic syndromes, extrarenal deformations and chromosomal abnormalities [8].

Opioids, especially morphine and fentanyl, rapidly cross the human placenta, affecting the fetus [9]. Typical opioids, such as fentanyl, exert their analgesic effects primarily by binding to µ-receptors in the central nervous system. These receptors are also present in the periphery, including the bladder. Activation of µ-receptors inhibits the release of neurotransmitters, including acetylcholine, which is leading to a decreased ability of the detrusor muscle to contract. In addition, opioids can inhibit the sensation of bladder fullness and increase bladder sphincter tone due to excessive sympathetic stimulation, resulting in a bladder outlet restraint [10, 11]. These effects are seen in human after high-dose opioid exposure. Propofol is a short acting hypnotic agent, which has not been associated with urinary retention [1]. Benzodiazepines, such as midazolam, act on gamma-aminobutyric acid (GABA) receptors in the central nervous system. Activation of GABA receptors may inhibit the release of neurotransmitters responsible for promoting bladder contractions, leading to a decrease in bladder activity [11].

Treatment of fetal megacystis or bladder rupture should be individualized, depending on underlying pathophysiology, gestational age, amount of fetal ascites and fetal condition. In the first or second trimester, vesicocentesis can be an effective treatment option for fetal megacystis [12]. Once fetal bladder rupture occurs watchful waiting is an option. Abdominal paracentesis of fetal ascites can be indicated if there are signs of abdominal compartment syndrome, such as pulmonary compromise in an early stage of pregnancy. If antenatal intervention is performed, it should only be indicated after careful consideration of the risk and benefits of the procedure, and should be performed in selected centers. Once fetal doppler velocimetry shows significant progressive deterioration, possibly caused by abdominal compartment syndrome in third trimester, delivery should be indicated.

## Conclusions

Fetal bladder rupture due to high-dose maternal opioid treatment is likely to be a rare phenomenon. To date, there is no clear proof of the connection between maternal opioid treatment and fetal bladder rupture. However, the association seems to strengthen as the number of case reports increase. More cases need to be described in order to estimate the prevalence. In addition, it could be useful to identify further details: for example, whether there is a critical time window in pregnancy for the negative effects of opioid therapy on the fetal bladder. In general, repeated ultrasound to detect fetal megacystis should be considered when maternal high-dose opioid-treatment is used. If a significant bladder distension develops, practicians should be alert.
